# Election of Medical Professors by Concours

**Published:** 1847-04

**Authors:** 


2. *Election of Medical Professors by Concours.*—Some two years ago we directed attention, by an article in this Journal, to the great good which would result to the profession of this country, and to Medical Colleges, by adopting the French mode of selecting Professors by *concours*—our views are yet unchanged.

We are right glad to see our friend of the Boston Med. and Surg. Journal, urging this mode of filling the vacant chair of anatomy in Harvard University. The following is what he says on the subject:

An important chair in the Medical College in Boston, is now well known to be vacant, by the resignation of Dr. Warren, who has sustained the professorship of Anatomy and Physiology, with distinguished ability and faithfulness, for an unusually long period. A query is running through the medical ranks in regard to his succesror. Who is the man?—is the question. The opinion prevails generally, that he has been long ago selected, and that the influence of one or two will determine any medical appointment at the University, against any competitor who might be so presumptuous as to aspire to a place of such professional value. Whether this is true or not we cannot pretend to decide. It is certain, however, that the fortunate person who obtains the appointment, in the ordinary way in which elections are made at all the academical and medical institutions in New England, will have the reputation of having been elevated by strong friends behind the screen;—and however meritorious he may be as a man, he will be contrasted with many greatly his superiors, but who had no friends at court, and whose attainments, therefore, and peculiar qualifications for lecturing acceptably and instructively, without great pillars of family strength or wealth, must edge their way through life, and market their knowledge at retail, instead of shining in conspicuous departments of science, for which, both by nature and education, they are pre-eminently qualified. Were the corporation of the University to throw the doors wide open, and invite the whole profession to contend honorably for the prize by concours, what a glorious triumph it would be for intellect! How probable it is that the election would fall on some individual whose transcendant powers are either unknown or not generally acknowledged by the public. And what an acquisition, too, would it be to a school, that should, in all coming ages, be the great and unrivalled medical focus of the Northern States.

It is quite unnecessary to particularize the character of the concours in France, or the effect the system has in developing the wonderful resources of the human mind. There is a prize to be gained worth contending for, when a professorship is there vacant. Men of comparative obscurity, having an opportunity to manifest their fitness for the duties, are permitted to exhibit their claims before a competent tribunal of judges, who are unswayed by those multiplied interests that are secretly made to bear upon a candidate's case, who silently glides into a fat postion, *a la New England.* There are said to be professorships in some medical schools in our country, where the endowment was made, provided the present incumbents were appointed to them. It was a regular piece of family economy, giving a relative a life annuity and college honors combined; in other words, without it, they would have been nothing in society, and even now, they are but make-weights or niche-fillers, like the baked monks of St. Bernard, for show in a faculty catalogue. Some persons ride through life on the shoulders of their friends, as Sindbad the Sailor did on the neck of the Old Man of the Sea, and look back upon the less fortunate of their fellow beings who are trudging on in the rear, indulging in feelings that are presumed to have agitated the benevolent Uncle Tobey, when he said to the fly, "go, poor devil, the world is large enough for thee and me." Being made, and making one's self, are very distinct affairs. History presents an unerring array of testimony to show that all the truly grand achievements in literature, science, and the arts, to say nothing of war, were accomplished by men who battled with adversity, and struggled against prejudices, but who at last triumphantly inscribed their own names on an imperishable tablet of universal fame.

No one conversant with the policy that usually actuates the managing spirits of institutions where profits or honor are at the disposal of a select board of gentlemen, supposes that the old scheme of suddenly making something out of nothing, will be readily abandoned. However disinterested some appointment may appear to the staring eyes of the spectator public, a large number of them, in the colleges of medicine in this country, have had their origin in an out-of-sight selfishness, difficult at all times to expose, but the trick is invariably detected in the sequel. We all have our favorites as well as relatives, and it is a weakness, perhaps, of humanity, that a sense of justice to the coming phalanxes of untaught students is lost sight of in the gratification of pushing a friend into a spot where an indirect advantage will accrue to us from his position.

All the hard sayings, often unjust surmises, inuendoes, and expressions of regret, which a numerous, jealous, ambitious profession may be supposed to manifest when, in important appointments, merit is smothered in a napkin, and brass is gravely declared, by the *Senatus Academicus*, to be gold, would be entirely obviated by the simple generous establishment of the system of election by concours. If the American journals would heartily advocate this excellent test of the qualifications of candidates for professorships, in the medical schools of the United States, a change in the present mode might be ultimately effected; and then, but never till that important revolution transpires, will the great body of our medical teachers, lecturers and professors, vie in true greatness and brilliancy with those in the schools of France.

